# Quantitative, Dynamic Detection of Neuronal Na^+^ Transients Using Multi-photon Excitation and Fluorescence Lifetime Imaging (FLIM) in Acute Mouse Brain Slices

**DOI:** 10.21769/BioProtoc.5175

**Published:** 2025-02-05

**Authors:** Sara Eitelmann, Karl W. Kafitz, Christine R. Rose, Jan Meyer

**Affiliations:** Institute of Neurobiology, Heinrich Heine University Düsseldorf, Düsseldorf, Germany

**Keywords:** ING-2, Sodium, Multi-photon imaging, Fluorescence lifetime imaging microscopy (FLIM), Hippocampus, Neuron, Chemical ischemia

## Abstract

Fluorescence lifetime imaging microscopy (FLIM) is a highly valuable technique in the fluorescence microscopy toolbox because it is essentially independent of indicator concentrations. Conventional fluorescence microscopy analyzes changes in emission intensity. In contrast, FLIM assesses the fluorescence lifetime, which is defined as the time a fluorophore remains in an excited state before emitting a photon. This principle is advantageous in experiments where fluorophore concentrations are expected to change, e.g., due to changes in cell volume. FLIM, however, requires collecting a substantial number of photons to accurately fit distribution plots, which constrains its ability for dynamic imaging. This limitation has recently been overcome by rapidFLIM, which utilizes ultra-low dead-time photodetectors in conjunction with sophisticated rapid electronics. The resulting reduction in dead-time to the picosecond range greatly enhances the potential for achieving high spatio-temporal resolution. Here, we demonstrate the use of multi-photon-based rapidFLIM with the sodium indicator ION NaTRIUM Green-2 (ING-2) for the quantitative, dynamic determination of Na^+^ concentrations in neurons in acute rodent brain tissue slices. We describe the loading of the dye into neurons and present a procedure for its calibration in situ. We show that rapidFLIM not only allows the unbiased determination of baseline Na^+^ concentrations but also allows dynamic imaging of changes in intracellular Na^+^, e.g., induced by inhibition of cellular ATP production. Overall, rapidFLIM, with its greatly improved signal-to-noise ratio and higher spatio-temporal resolution, will also facilitate dynamic measurements using other FLIM probes, particularly those with a low quantum yield.

Key features

• RapidFLIM of the sodium indicator ING-2 enables the intensity-independent recording of neuronal Na^+^ transients at unparalleled full frame rates of 0.5–1 Hz.

• RapidFLIM is essentially independent of dye concentrations and therefore not affected by dye bleaching.

• Full in situ calibrations enable the quantification of intracellular Na^+^ changes at high spatio-temporal resolution.

• RapidFLIM of ING-2 allows unbiased determination of cellular Na^+^ loading also in conditions of strong cell swelling.

## Graphical overview


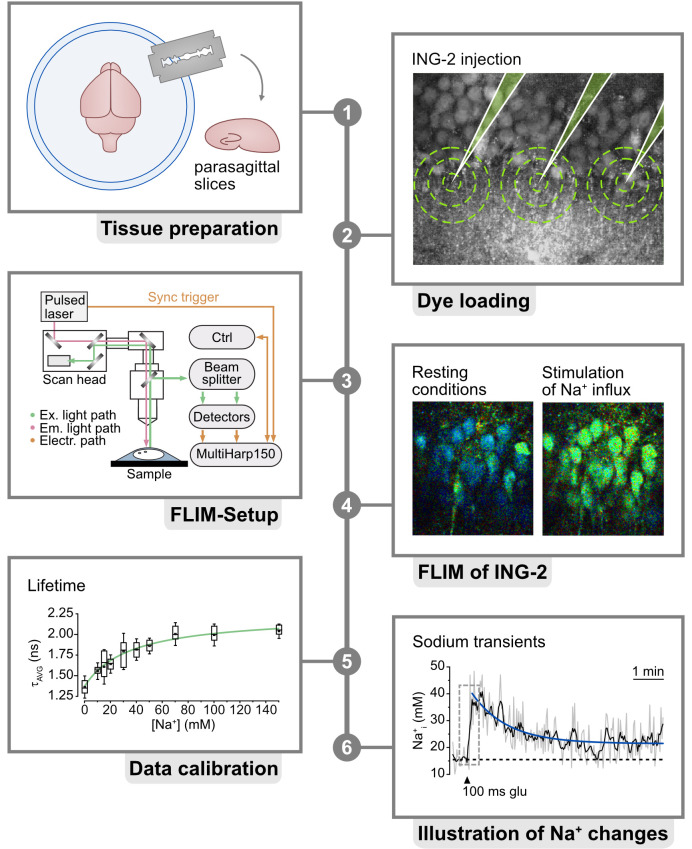


## Background

Fluorescence lifetime imaging microscopy (FLIM) has emerged as an important technique for gaining a deeper understanding of ion concentration dynamics inside cells of the central nervous system [1–4]. In contrast to widely used intensity-based imaging, FLIM provides information based on the fluorescence lifetime, which is the duration that a fluorophore remains in an excited state before photon emission [5]. For many chemical indicator dyes, such as the widely used calcium indicator Oregon Green 488 BAPTA-1 [1,6], this excited state lifetime is correlated to ion binding. FLIM therefore enables a direct, quantitative readout of ion concentrations, provided that appropriate calibrations are carried out. Importantly, the fluorescence lifetime is largely independent of the concentration of the indicator dyes. This is particularly advantageous in experimental conditions where fluorophore concentrations change, e.g., due to bleaching or changes in cell volume [7]. However, traditional FLIM is subject to so-called dead-time artifacts, which greatly reduce the number of usable photons [8]. Dead-time artifacts result in the occurrence of seemingly shorter lifetimes when the photon count rates exceed a specific threshold, typically between 1% and 5% of the laser repetition rate. This necessitates the use of long collection times, of up to over one minute, to adequately fit the decay of the photon arrival times [9]. The resulting decrease in temporal resolution restricts the ability of FLIM for fast dynamic imaging.

The development of rapidFLIM, which reduces the dead time from approximately 100 ns to roughly 0.7 ns, has overcome this technical issue, allowing much faster repetition rates [10,11]. This enables the FLIM-based dynamic recording of transient changes in ion concentrations that are characteristic of cells of the nervous system, such as fluctuations in the intracellular Na^+^ concentration [3]. Na^+^ is integral to brain function, exerting a profound influence on both neurons and astrocytes through a multitude of cellular mechanisms. Disruptions in Na homeostasis result in altered neuronal excitability and impaired neurotransmission and contribute to the development of neurological disorders [12,13]. FLIM measurements of the intracellular Na^+^ concentration can be performed using the chemical indicator dye ION NaTRIUM Green-2 (ING-2), which shows an approximately 1–1.5-fold change in fluorescence lifetime upon Na^+^ binding [3,14,15]. ING-2 offers a relatively broad two-photon cross-section, allowing excitation within the range of 750–1,000 nm, while emission peaks between 500 and 600 nm [14,15]. Recently, we demonstrated for the first time that multi-photon-based, rapidFLIM enables dynamic imaging of Na^+^ transients in neurons in acute hippocampal tissue slices at so far unprecedented spatio-temporal resolution. Notably, this also allowed unbiased quantitative measurement of changes in neuronal Na^+^ induced by chemical ischemia, i.e., in conditions of strong cell swelling [3]. The present protocol describes the procedures for rapidFLIM for the quantitative recording of intracellular Na^+^ with ING-2, including the procedures for in situ calibration.

## Materials and reagents

**Biological materials**

1. Balb/c mice bred by the Animal Care and Use Facility of the Heinrich Heine University Düsseldorf. Alternatively, other suitable rodent models can be used (e.g., Janvier, BALB/cJRj)

**Reagents**

1. Sodium chloride (NaCl) (Roth, catalog number: 3957)

2. Potassium chloride (KCl) (Roth, catalog number: 6781.1)

3. Calcium chloride dihydrate (CaCl_2_) (Fluka/Honeywell, catalog number: 31307)

4. Magnesium chloride hexahydrate (MgCl_2_) (Roth, catalog number: 2189.1)

5. Di-sodium hydrogen phosphate (Na_2_HPO_4_) (Roth, catalog number: 4984.1)

6. Sodium hydrogen carbonate (NaHCO_3_) (Applichem, catalog number: 131965)

7. Glucose (Caelo, catalog number: 2580)

8. 2-[4-(2-Hydroxyethyl)-1-piperazine]ethanesulfonic acid (HEPES) (Fisher, catalog number: BP310-500)

9. Magnesium sulfate (MgSO_4_) (Sigma-Aldrich, catalog number: 230391)

10. D-Gluconic acid sodium salt (Na-gluconate) (Sigma-Aldrich, catalog number: G9005-500G)

11. Gluconic acid potassium salt (K-gluconate) (Roth, catalog number: 2621)

12. Monensin sodium salt (Alfa Aesar, catalog number: J61669)

13. Potassium iodide (Thermo Fisher, catalog number: A12704.18)

14. Erythrosin B analytical standard, ≥ 98.0% (HPLC) (Erythrosin B) (Sigma-Aldrich, catalog number: 87613)

15. Gramicidin (Sigma-Aldrich, catalog number: G5002)

16. Ouabain octahydrate (Merck, catalog number: 4995)

17. ION NaTRIUM Green^TM^-2 acetoxymethyl ester (ING-2 AM) (Mobitec, catalog number: 2011F)

18. Pluronic^TM^ F-127 (Pluronic) (Invitrogen, catalog number: P6867)

19. Sulforhodamine 101 (SR101) (Sigma-Aldrich, catalog number: S7635-50MG)

20. Dimethyl sulfoxide (DMSO) (Sigma-Aldrich, catalog number: D8418)

**Solutions**

1. Artificial cerebrospinal fluid (standard ACSF) (see Recipes)

2. Modified ACSF for preparation of brain slices (preparation ACSF) (see Recipes)

3. HEPES-based ACSF (see Recipes)

4. ACSF for calibration (see Recipes)

5. SR101 stock solution (see Recipes)

6. ING-2 AM stock solution (see Recipes)

7. Bolus-loading solution (see Recipes)

8. Instrument response function solution (IRF solution) (see Recipes)

**Recipes**

**1. Standard ACSF**

**Table:** 

Reagent	Final concentration in double-distilled water
NaCl	130 mM
KCl	2.5 mM
CaCl_2_	2 mM
MgCl_2_	1 mM
NaH_2_PO_4_	1.25 mM
NaHCO_3_	26 mM
Glucose	10 mM

Adjust pH to 7.4 by bubbling with carbogen (95% O_2_ and 5% CO_2_).

The recommended overall volume is 1,000 mL.

**2. Preparation ACSF**

**Table:** 

Reagent	Final concentration in double-distilled water
NaCl	130 mM
KCl	2.5 mM
CaCl_2_	0.5 mM
MgCl_2_	6 mM
NaH_2_PO_4_	1.25 mM
NaHCO_3_	26 mM
Glucose	10 mM

Adjust pH to 7.4 by bubbling with carbogen (95% O_2_ and 5% CO_2_).

The recommended overall volume is 1,000 mL.

**3. HEPES-based ACSF**

**Table:** 

Reagent	Final concentration in double-distilled water
NaCl	125 mM
KCl	3 mM
CaCl_2_	2 mM
MgSO_4_	2 mM
NaH_2_PO_4_	1.25 mM
HEPES	25 mM
Glucose	10 mM

Adjust pH to 7.4 by titration with NaOH.

The recommended overall volume is 100 mL.

**4. ACSF for calibration**

*Note: Prepare calibration solutions with different sodium concentrations (e.g., 0, 10, 50, and 100 mM) by adjusting the concentration of K^+^, maintaining osmolarity. Solutions need to be prepared freshly every day.*

**Table:** 

Reagent	Final concentration in double-distilled water
Na^+^ + K^+^	150 mM
Cl-	30 mM
Gluconic acid	120 mM
HEPES	10 mM
MgSO_4_	2 mM
CaCl_2_	2 mM
Gramicidin	3 μM
Monensin	10 μM
Ouabain	1 mM

Adjust pH to 7.4 by titration with KOH.

The recommended overall volume is 500 mL for each.

**5. SR101 stock solution**

**Table:** 

Reagent	Final concentration	Quantity or Volume
SR101	1.5 mM	-
Double-distilled H_2_O	-	1 mL

**6. ING-2 AM stock solution**

**Table:** 

Reagent	Final concentration	Quantity or Volume
ING-2 AM	1 mM	-
Pluronic	20 mg/mL	-
DMSO	-	46.12 μL

**7. Bolus-loading solution**

**Table:** 

Reagent	Final concentration	Quantity or Volume
ING-2 AM stock solution	125 μM	2 μL
HEPES-based ACSF	-	14 μL

**8. IRF Solution**

*Note: Dissolve potassium iodide in H_2_O to saturation. Then, add 0.5 mg of Erythrosin B per 5 mL of H_2_O.*

**Table:** 

Reagent	Final concentration	Quantity or Volume
Potassium iodide	Until saturation	-
Double-distilled H_2_O	-	5 mL
Erythrosin B	-	0.5 mg

**Laboratory supplies**

1. Cyanoacrylate glue (e.g., Superflex gel, UHU)

2. Razor blades (e.g., razor blades classic, Wilkinson sword)

3. Scalpel blades (e.g., Heinz Herenz)

4. Filter paper (e.g., plain disc filter paper, Lab Logistics Group GmbH)

## Equipment

**Figure 1.**
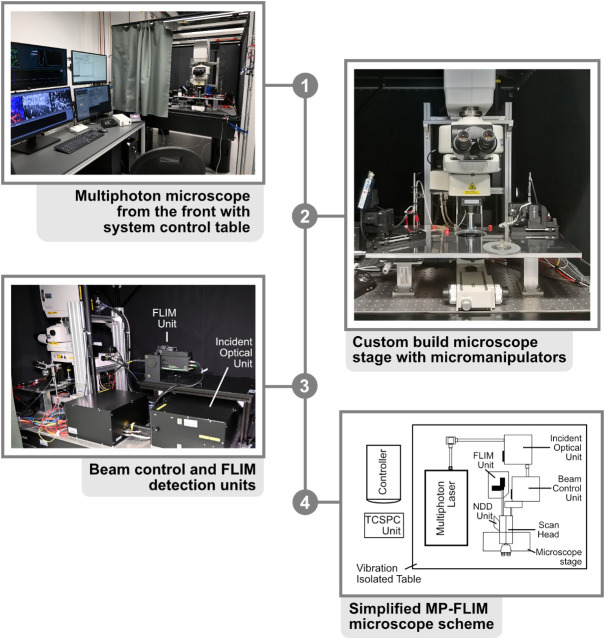
Multiphoton microscope setup. 1. Front view of the Nikon A1R MP with system control table. All software necessary for acquisition is started on the adjacent workstations and visible on the respective monitors. **2.** Close-up of the microscope stage, with the slice chamber and micromanipulators present in the middle. **3.** Backside of the Nikon A1R MP with customized emission port. FLIM unit and incident optical unit are visible in the middle. **4.** Simplified scheme of the beam path. In the case of the A1R MP, the excitation beam is directed from the laser to the incident box and optical unit, where it is guided to the scan head of the microscope. After exciting the probe, the emission beam is directed to the FLIM detectors with as few protrusions (e.g., mirrors and filters) as possible, for maximal photon collection efficacy.

1. Microtome (e.g., Campden Instruments, model: Campden 7000smz-2)

2. Micropipette puller (e.g., Narishige, model: PC-100)

3. Micromanipulator (e.g., Luigs & Neumann, model: LN Junior)

4. Pressure application device for dye injection (e.g., NPI Electronic GmbH, model: PDES)

5. Standard harp slice grids (e.g., ALA Scientific, model: HSG-5A or Warner SHD 41/10)

6. Photon count rate-optimized and custom-modified multiphoton laser imaging system (Figure 1) (a) with a high-intensity pulsed laser source (b) and time-correlated single photon counting (TCSPC) unit for FLIM data acquisition (c), e.g.,

a. Nikon A1MP (Nikon Europe) (Figure 1, steps 1–3)

b. MaiTai DeepSee (Newport Spectra-Physics GmbH, MKS) (Figure 1, step 4)

c. Multiharp 160, PicoQuant GmbH (Figure 1, step 3–4)

Custom modifications mainly include hardware and optical adaptations in the microscope, the NDD stage, and the connection between the microscope and the FLIM detector unit to prevent the reduction of the emission signal intensity going to the FLIM detection unit. These kinds of modifications require the help of both mechanical and electronic workshops and the support of the microscope manufacturer.

## Software and datasets

1. NIS Elements Advanced Research (5.21, 08/28/2024, Nikon)

2. SymPhoTime 64 (2.8, 08/28/2024, PicoQuant)

3. OriginPro^®^ (2021, 08/28/2024, OriginLab)

4. Microsoft Excel (Office 2021, 08/28/2024, Microsoft Corporation)

5. Affinity Designer [1.10, 08/28/2024, Serif (Europe) Ltd.]

## Procedure

**A. Preparation of acute hippocampal brain slices**

**Figure 2.**
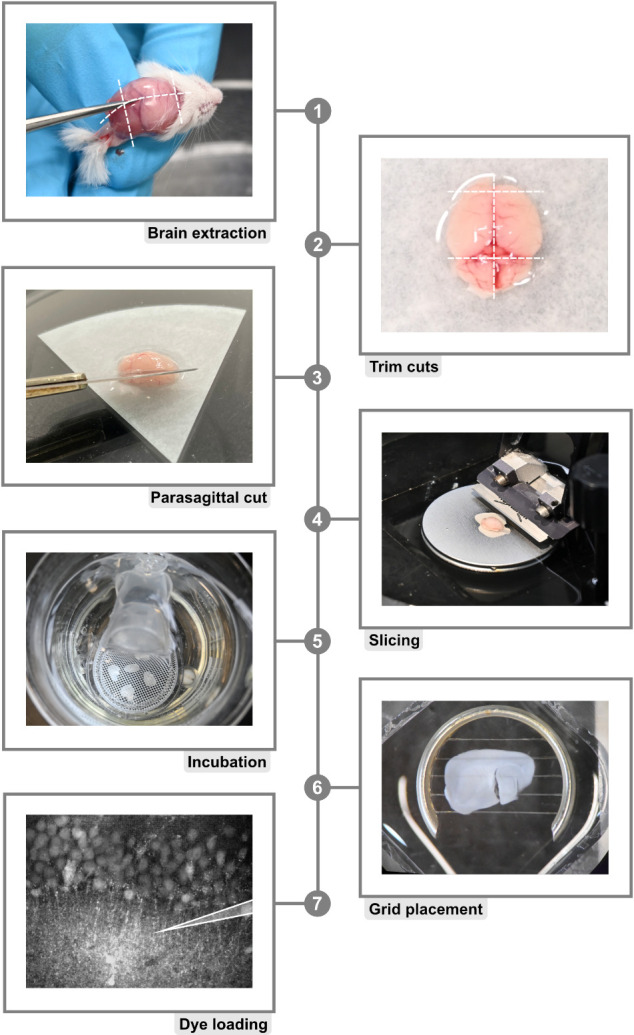
Protocol for acute brain slice preparation. 1. Brain isolation starts by cutting along the illustrated dashed lines. Note: It is necessary to work fast to minimize the time period in which the brain is not submerged in ice-cold ACSF (see below). Always use maximum care to prevent damage to the tissue probes. **2.** Excess tissue is removed with additional trimming cuts as depicted by dashed lines. **3.** Parasagittal trimming is achieved by cutting a single hemisphere at a 45° angle. **4.** The hemisphere is glued to the vibratome cutting stage and placed in the buffer tray filled with ice-cold, carbonated modified ACSF. **5.** Freshly cut slices are placed onto a mesh positioned in a beaker filled with modified, ice-cold ACSF. **6.** Just before starting experiments, slices are positioned in the experimental chamber and secured with a fine grid. **7.** Bolus-loading of hippocampal CA1 region.

1. To obtain acute brain slices, mice older than postnatal day 14 are anesthetized with CO_2_ and subsequently rapidly euthanized by decapitation (for a detailed explanation of animal policies, see also the section Ethical considerations) (Figure 2, step 1). The procedure can also be used for adult animals or animals younger than P14. Note, however, that different protocols for anesthesia may apply.

2. Following decapitation, remove the brain rapidly and place it in a Petri dish containing ice-cold preparation ACSF. To maintain a pH of 7.4, ACSF must be continuously bubbled with carbogen (95% O_2_ and 5% CO_2_) (Figure 2, step 2).

3. After the separation of the hemispheres, make an additional incision in a parasagittal orientation (approximately 1.5 mm thick). Affix the tissue block containing the hippocampus with glue to the cutting stage of the microtome at the parasagittal incision. Place the cutting stage into the microtome and cool (ideally with a non-dissolving cooling element) (Figure 2, step 3).

4. Prepare hippocampal slices with a thickness of 250 μm, keeping the tissue constantly submerged in ice-cold preparation ACSF (Figure 2, step 4).

5. Transfer the slices very gently to ACSF containing 1 μM SR101 at 34 °C for 20 min, during which they will take up the astrocyte marker. Following this, incubate the slices at 34 °C for a further 10 min in standard ACSF. Slices can be used for experiments for approximately 5–7 h if kept at room temperature (Figure 2, step 5).

**B. Bolus-loading of ING-2 into hippocampal brain slices**

*Note: This technique was initially developed for in vivo loading of dyes into brain cells [16]. It has since been adapted for use with acute brain slices and is employed by numerous research laboratories, including our own.*

**Figure 3.**
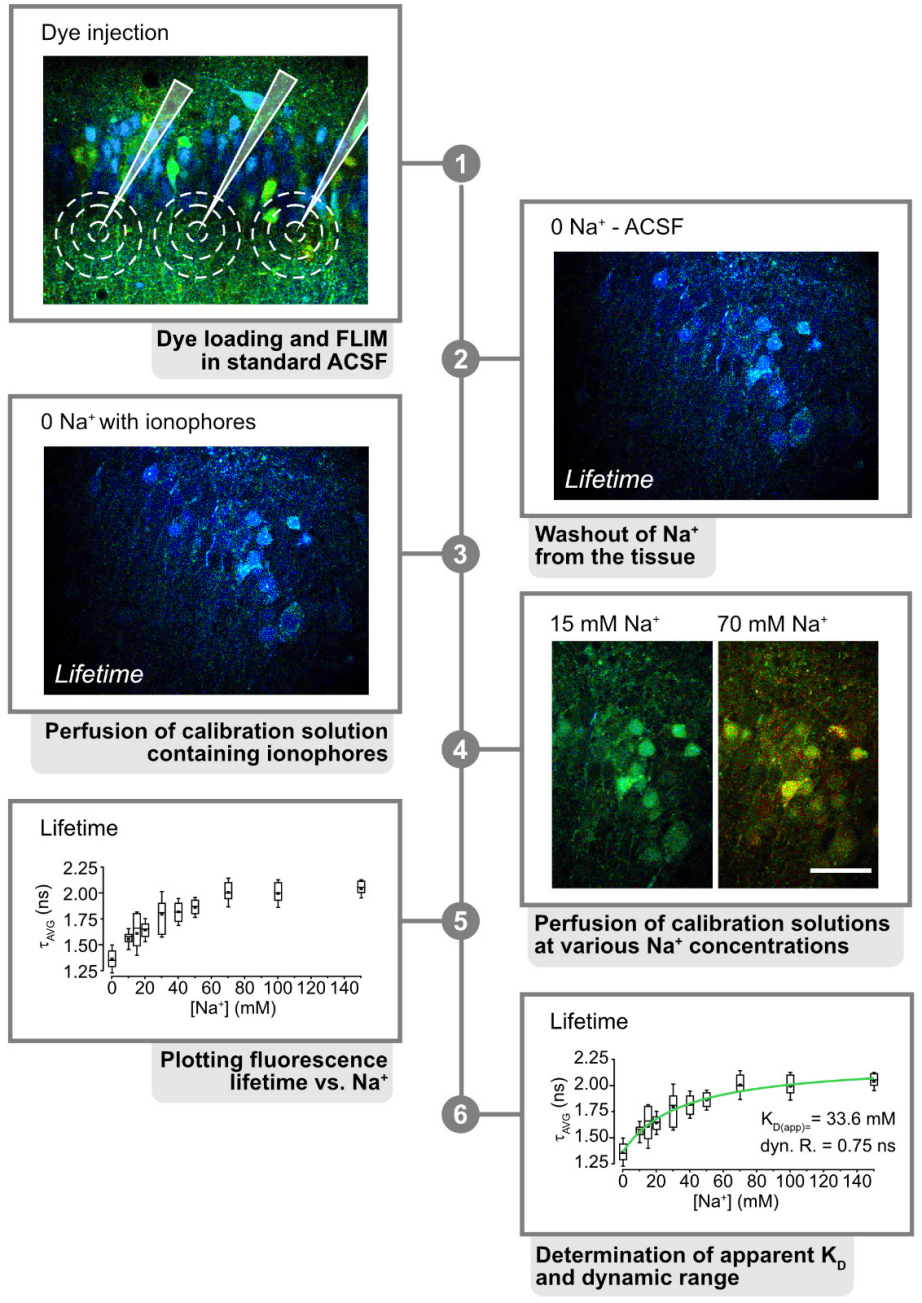
Calibration process. 1. Inject ING-2 at the region of interest. Here, a fluorescence lifetime image of the CA1 region of a hippocampal tissue slice is shown. Pipettes and approximate areas, dye-loaded by one injection, are indicated in white. **2.** Perfuse the slice with Na^+^-free saline to wash out extracellular Na^+^. **3.** Perfuse with 0 Na^+^ calibration solution containing ionophores to equilibrate intra- and extracellular Na^+^. **4.** Continue perfusion with calibration solutions at different Na^+^ concentrations. **5.** Plot the resulting lifetimes against Na^+^ using appropriate software (e.g., Origin Pro). **6.** Fit the resulting data to determine the apparent K_D(app)_ and dynamic range of the dye. Modified and taken from [3].

1. Prepare pipettes for dye injection (tip diameter of approximately 1 μm and a resistance of 1–3 MΩ) from fire-polished borosilicate glass capillaries using a standard micropipette puller.

2. Place a hippocampal brain slice into a microscope chamber and fix it with a “harp” (e.g., ALA Scientific HSG-5A) (Figure 2, step 6). After placing the chamber in your imaging system, constantly perfuse the brain slice with standard ACSF (typical perfusion rate is 2 mL/min).

3. Load an injection pipette with bolus-loading solution (see Recipes) and attach the pipette to the pressure application device.

4. Position the pipette with care and the help of a micromanipulator into your region of choice in the tissue (Figure 2, step 7). Dye injection should be close to the cellular region you are interested in, usually in the same field of view. For loading of somata of neurons in the CA1 pyramidal area, the dye injection pipette should be positioned, e.g., in the stratum radiatum, just below the CA1 pyramidal layer (Figure 3, step 1). Apply ~1.5 psi for ~5 s to inject the dye into the tissue. This process should be repeated until the entire field of view is covered (Figure 3, step 1).

5. Wait for 30–45 min to engage intracellular de-esterification and wash off excess dye from the extracellular space, constantly perfusing the brain slice with standard ACSF.

**C. Fluorescence lifetime imaging microscopy of ING-2 and multi-photon excitation**

1. Choose suitable parameters for imaging for your given imaging system and the specific dye used. With multiphoton excitation, ING-2 can be excited over a broad wavelength spectrum from 740 to 1,100 nm. Typical parameters for ING-2 used in our experiments are an excitation wavelength of 840 nm, an image size of 512 × 512 pixels, and an imaging frequency of 1 Hz.

*Note 1: The whole setup should be tuned for maximum photon efficacy. The more photons that can be recorded, the better the spatio-temporal resolution will be. Temporal resolution is always relative, as the recorded frames can be binned afterward. However, a minimum number of photons (for Na^+^-sensitive dyes 15–20 photons per pixel and frame) need to be recorded for proper lifetime calculation.*

*Note 2: The setup process before every experiment is crucial to obtain high-quality results. Make sure that the laser power under the objective does not exceed 5 mW. Use appropriate emission filters and dichroic mirrors to ensure optimal detection conditions. For ING-2, emission can be collected above 560 nm wavelength.*

2. Measure the instrument response function (IRF) of your microscope with the IRF solution (see Recipes) using comparable photon counts as during experimental conditions (e.g., same laser power).

3. Select your region of interest in your tissue preparation.

**Critical:** Make sure that your tissue sample is healthy and that many viable neurons are present. Injured neurons are usually characterized by their swollen cell bodies. Moreover, dendrites may be distorted and not be clearly visible.

4. Record an image (e.g., an average of 30 frames) in standard ACSF to check initial conditions.

5. Start a time series in standard ACSF and record fluorescence lifetime in baseline conditions (e.g., for 30 s).

6. Next, record a time series performing your manipulation of choice to test if this results in a change in fluorescence lifetimes (and intracellular Na^+^). As a positive control, bath application of glutamate (e.g., 1 mM glutamate for 10 s) causes reliable and prominent intracellular Na^+^ signals in rodent hippocampal slices [17]. Moreover, bath application of inhibitors of cellular ATP production induces long-lasting and large increases in intracellular Na^+^ in the same preparation [2,3,18]. Alternatively, substances can be focally applied to a soma or a dendrite. This is usually done with a fine glass pipette (comparable to the ones used in section B for bolus application but with a smaller tip diameter) that is filled with the substance of choice (e.g., 1 mM glutamate). The application duration can be a lot shorter in comparison to bath applications and is usually below 1 s [3,19,20].

7. Check if the changes induced by the manipulation are reversible, i.e., if fluorescence lifetimes recover to the initial baseline levels. After the lifetimes have returned to initial values for at least 30 frames, stop the time series.

8. Take another image (e.g., again an average of 30 frames) to compare starting and finishing conditions in standard ACSF.

**D. Calibrating changes in lifetime to changes in Na^+^**

*Note: The general calibration routine for Na^+^-sensitive indicator dyes was described in detail by Rose and Ransom [21] for cultured astrocytes. It was later adapted for acute hippocampal brain slices and widefield and confocal as well as multi-photon microscopy [3,22,23]. This protocol was first used for FLIM by Meyer et al. [2].*

1. Prepare calibration solutions with different Na^+^ concentrations, ranging from unsaturated (0 mM Na^+^) to saturated (>100 mM Na^+^) concentrations.

**Caution:** Select a frame rate and laser power, which enables the collection of sufficient photons under all conditions.

2. Start perfusing a dye-loaded tissue slice (Figure 3, step 1) with standard ACSF and then switch to 0 mM Na^+^ solution *without* the ionophores and ouabain to wash out Na^+^ from the tissue (Figure 3, step 2).

3. Record the resulting changes in fluorescence lifetime.

4. Then, perfuse the slice with 0 mM Na^+^ solution *containing* the ionophores and ouabain to promote washout of Na^+^ from the cells and to enable equilibration of Na^+^ between intra- and extracellular space (Figure 3, step 3).

5. Switch to calibration salines containing different Na^+^ concentrations (e.g., 10, 20, 30, 50, and 100 mM), again recording changes in fluorescence lifetime resulting from the changes in Na^+^ (Figure 3, step 4).

*Note: Always wait before switching to the next concentration until a stable plateau has been reached. You might need to adjust the focus of your microscope during the calibration process.*

6. Plot the changes in lifetime against Na^+^ and use a Michaelis–Menten equation to fit the data points and to determine the apparent K_d_ and the dynamic range/saturation of the dye used (Figure 3, step 5).

7. The resulting fit will also allow you to directly convert fluorescence lifetime values obtained during different experiments into Na^+^ concentrations (Figure 3, step 6).

## Data analysis

1. Start by loading an image into your analysis software.

2. Calculate the IRF using the measurement of the IRF solution (see step C2).

3. Select the region of interest you would like to analyze (e.g., a single, neuronal cell body).

4. Determine the lifetime distribution from the chosen region of interest.

5. Fit a mono- or multi-exponential decay to your measured fluorescence lifetime distribution.

*Note: Finding optimal fitting parameters can be a tedious task. Decays can be fitted, mono-, bi-, or multi-exponential depending on the used indicators. When fitting with more than one exponent, using fixed exponents can increase the signal-to-noise ratio.*

6. Export the fitted results for data visualization and basic analysis in a suitable program (e.g., Excel, Origin Pro).

7. Use an appropriate program for the visualization of the data in the form of figures (e.g., Affinity Designer).

## Validation of protocol

This protocol or parts of it has been used and validated in the following research articles:

• Meyer et al. [2]. Quantitative Determination of Cellular [Na^+^] By Fluorescence Lifetime Imaging With CoroNaGreen. *Journal of General Physiology*.

• Meyer et al. [3]. Rapid Fluorescence Lifetime Imaging Reveals That TRPV4 Channels Promote Dysregulation of Neuronal Na^+^ in Ischemia. *Journal of Neuroscience*.

**Figure 4.**
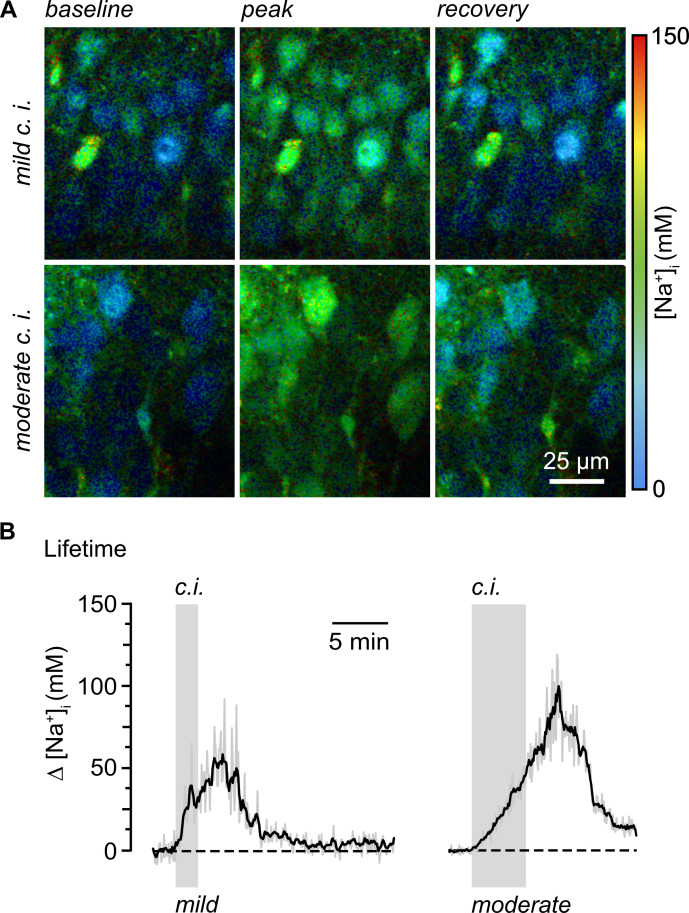
Quantitative, dynamic measurement of Na^+^ loading in hippocampal neurons using multi-photon excitation and rapid FLIM with ING-2. A. Color-coded fluorescence lifetime images (30 s temporal binning) of the intracellular Na^+^ depicting the CA1 pyramidal cell layer of an ING-2-loaded acute hippocampal tissue slice. Right: color code. Top left: image showing intracellular Na^+^ at baseline conditions; middle: change in Na^+^ upon mild chemical ischemia (2 min of perfusion with 2 mM NaN_3_ and 5 mM 2-Desoxyglucose); right: recovery taken ~20 min after starting perfusion with inhibitors. Bottom: similar illustration, demonstrating the neuronal Na^+^ loading upon moderate chemical ischemia (same solution as before but with 5 min of application). **B.** Changes in intracellular Na^+^ induced by mild (2 min; left) and moderate (5 min; right) chemical ischemia (indicated by the gray boxes) reported by rapidFLIM in two different experiments. Gray traces: individual cells; black traces: averages of all neurons analyzed in one particular experiment (n = 35 and 40, respectively). Note that data points were constrained to the minimum/maximum value of the calibration (0 and 150 mM Na^+^). Images modified and taken from [3].

These two publications illustrate and describe the above-mentioned in situ calibrations in detail, also providing approaches for dye calibration in vitro. Meyer et al. [3] described rapidFLIM in rodent brain tissue slices, also demonstrating that this technique enables reliable, unbiased determination of baseline Na^+^ concentrations in CA1 pyramidal neurons. Moreover, Meyer et al. [3] presented different manipulations to induce reversible changes in neuronal Na^+^ and their recording by rapidFLIM. Figure 4 shows that inhibition of cellular metabolism by perfusing tissue slices with glucose-free saline containing inhibitors of glycolysis and oxidative phosphorylation results in large increases in Na^+^ in CA1 pyramidal neurons.

## General notes and troubleshooting

**General notes**

1. All solutions for experiments and preparation should be prepared freshly on the day of use. Standard and preparation ACSF need to be bubbled with carbogen for at least 30 min before use to ensure a correct pH of 7.4.

2. Acute brain slices need to be treated with utmost care. Bending and other mechanical stress result in damaged cells.

3. As optimizing the experimental setup is critical to obtain optimal photon counts, it is recommended to test imaging parameters in an in vitro environment first before using acute brain slices.

4. Most ion-sensitive dyes are diluted in 20% Pluronic/DMSO. Pluronic is sensitive to freeze and unfreeze cycles, which therefore should be avoided.

5. The total number of collected photons directly correlates to the quality of your measurements. Temporal resolution does directly increase with an increased number of collected photons. The measurement setup should therefore be tweaked for maximum photon collection. This can be achieved by using as few mirrors and filters as possible and using the highest possible grade of optical components.

6. Many manufacturers offer dye spectra and calibration curves obtained in vitro (in the cuvette). Note that most chemical ion indicators change their properties when loaded inside cells. Therefore, in situ calibration is highly recommended.

**Troubleshooting**

Problem 1: Photon counts are generally too low.

Possible cause: Something might be blocking the light path.

Solution: Check the microscope for any possible obstructions or optical deficits (c.f. problem 4).

Problem 2: Lifetimes are generally too short.

Possible cause: You might be running into *photon pileup*.

Solution: Modern detectors with lower dead times show nearly no photon pileup effects. Either change detectors or reduce laser power to more adequate count rates.

Problem 3: Staining is too weak.

Possible cause 1: Your staining solution might be old, or ACSF may have been sucked up into the tip of the pipette.

Solution: Prepare a fresh staining solution and control the pre-pressure of your application device.

Possible cause 2: Your preparation is not viable; slices may contain many dead cells.

Solutions: Try to minimize cell damage by treating the tissue with utmost care. Re-adjust your vibratome to minimize vertical blade movements. Pay attention to reducing preparation durations as much as possible.

Problem 4: The photon counts are insufficient for calculating a lifetime, and the statistical fit is inadequate.

Possible cause: You might not have enough photons per frame.

Solutions: Either increase laser power or increase the number of binned frames. Try to optimize the optical configuration of your microscope system by decreasing the number of optical components and increasing the optical quality of the components.

Problem 5: Lifetimes are different than expected and/or not changing upon stimulation.

Possible cause: Crosstalk of autofluorescence or a second fluorescent dye.

Solution: If you are using a second dye for cell identification, check for possible crosstalk and bleed-through. Also, check the autofluorescence of your specimen for possible lifetime artifacts. Crosstalk needs to be eliminated completely to be able to get a proper and stable lifetime readout.
